# Supplementary material for: The Systems Biology Research Tool: evolvable open-source software
Source: BMC Syst Biol. 2008 Jun 29;2:55. doi: 10.1186/1752-0509-2-55 (PMC2446383; doi:10.1186/1752-0509-2-55)
Supplement: Additional file 1 — SBRT Archive. An archive of the current version of the Systems Biology Research Tool. [file 1752-0509-2-55-S1.zip › sbrt-1.4.0/doc/developers_guide/api/sbrt/shell/mng/fba/CdHarFluxVectorManager.html]

CdHarFluxVectorManager


|  |  |  |
| --- | --- | --- |
| **Overview** \| **Package** \| **Class** \| **Use** \| **Tree** \| **Deprecated** \| **Index** \| **Help** | |  |
| **PREV CLASS**   **NEXT CLASS** | **FRAMES**    **NO FRAMES**     **All Classes** |  |
| SUMMARY: NESTED \| FIELD \| CONSTR \| METHOD | DETAIL: FIELD \| CONSTR \| METHOD |  |


---


## sbrt.shell.mng.fba Class CdHarFluxVectorManager

```
java.lang.Object
  sbrt.shell.mng.AbstractProcessManager
      sbrt.shell.mng.fba.FbaManager
          sbrt.shell.mng.fba.RandomFluxVectorManager
              sbrt.shell.mng.fba.CdHarFluxVectorManager
```

**All Implemented Interfaces:**: KernelProcessManager<java.lang.String,java.lang.String>, ProcessManager<java.lang.String,java.lang.String>

---

``` public final class CdHarFluxVectorManager extends RandomFluxVectorManager ```

This class is used to manage FBA coordinate direction hit-and-run random flux
vector experiments.

**Author:**
:   This class was written and documented by Jeremiah Wright while in the
    Wagner lab.

---

| **Field Summary** | |
| --- | --- |
| `static UnmodifiableSet<java.lang.String>` | `optionalKeywords`             The set of keywords required to be present in the `ManagerInput` objects supplied to instances of this class. |
| `static UnmodifiableSet<java.lang.String>` | `requiredKeywords`             The set of keywords required to be present in the `ManagerInput` objects supplied to instances of this class. |

**Fields inherited from class sbrt.shell.mng.fba.RandomFluxVectorManager**

`ALGORITHM`

**Fields inherited from class sbrt.shell.mng.fba.FbaManager**

`CONSTRAINT_TOLERANCE, DEFAULT_CONSTRAINT_TOLERANCE, FLUX_VECTOR_FILE, FLUX_VECTOR_FILE_NAME, RXN_CATALYST_FILE, RXN_FILE, SYSTEM_SOLUTION_FILE`

**Fields inherited from class sbrt.shell.mng.AbstractProcessManager**

`COMPLETED, DATA_HEADERS, DEV_NULL, ELAPSED_TIME, FILE_FORMAT, INPUT_FILE, INPUT_FILE_FORMAT, INPUT_FILE_NAME_FILE, ITERATIONS, OUTPUT_FILE_FORMAT, OUTPUT_FILE_NAME, OUTPUT_FILE_NAME_FILE, PERCENT, PERCENT_COMPLETED, SEED, ZERO_CUTOFF`


**Constructor Summary**

`CdHarFluxVectorManager()`


| **Method Summary** | |
| --- | --- |
| `void` | `execute(java.io.PrintWriter out)`             Executes the process this process manager controls, and writes the status or results of this process to the provided print writer. |
| `static CdHarFluxVectorComputer` | `getComputer(ManagerInput<java.lang.String,java.lang.String> input, java.io.PrintWriter out)`             Creates and returns a coordinate-direction hit-and-run flux vector computer using the provided input. |
| `static CdHarFluxVectorComputer` | `getComputer(ManagerInput<java.lang.String,java.lang.String> input, java.io.PrintWriter out, IrrevRxnFluxome fluxome, double tolerance)`             Creates and returns a coordinate-direction hit-and-run flux vector computer using the provided input. |
| `static java.util.Map<java.lang.String,java.lang.Double>` | `getFluxVector(ManagerInput<java.lang.String,java.lang.String> input, java.io.PrintWriter out, Fluxome fluxome, double tolerance)`             Opens the file denoted by `CdHarManager.INITIAL_POINT_FILE` in the provided manager input and returns the flux vector it contains. |
| `static MapOutputFile<java.lang.String,java.lang.Double>` | `getMultiFluxVectorOutputFile(ManagerInput<java.lang.String,java.lang.String> input, java.io.PrintWriter out, Fluxome fluxome)`             Returns an empty output file that is suitable for storing multiple flux vectors. |
| `UnmodifiableSet<java.lang.String>` | `getOptionalKeys()`             Returns the set of keywords the provided `ManagerInput` object does not necessarily have to contain. |
| `CdHarFluxVectorProcess` | `getProcess()`             Returns the process this process manager controls. |
| `UnmodifiableSet<java.lang.String>` | `getRequiredKeys()`             Returns the set of keywords the provided `ManagerInput` object must contain. |
| `static ApplicationException` | `interpret(DimensionException e)`             Returns an `ApplicationException` whose message describes the error that ocurred. |
| `static ApplicationException` | `interpret(ExceededLimitException e)`             Returns an `ApplicationException` whose message describes the error that ocurred. |
| `static ApplicationException` | `interpret(InfeasibleSolutionException e)`             Returns an `ApplicationException` whose message describes the error that ocurred. |
| `static ApplicationException` | `interpret(InvalidFluxVectorException e)`             Returns an `ApplicationException` whose message describes the error that ocurred. |
| `static ApplicationException` | `interpret(java.lang.RuntimeException e)`             Attempts to interpret the provided RuntimeException. |
| `static ApplicationException` | `interpret(UnboundedVariableException e)`             Returns an `ApplicationException` whose message describes the error that ocurred. |
| `void` | `setInput(ManagerInput<java.lang.String,java.lang.String> input, java.io.PrintWriter out)`             Uses the provided input to prepare this process manager for execution. |

**Methods inherited from class sbrt.shell.mng.fba.RandomFluxVectorManager**

`getConstraints, getConstraints, getFluxome, getFluxVector`

**Methods inherited from class sbrt.shell.mng.fba.FbaManager**

`getCatalysts, getCatalysts, getConstraintTolerance, getConstraintTolerance, getFluxome, getFluxome, getFluxome, getFluxome, getFluxomeSolution, getMultiFluxVectorOutputFile, getRxnNameExprFormat, getRxnNameHeaders, getRxnNameHeaders, getSingleFluxVectorOutputFile, getSingleFluxVectorOutputFile, printFluxomeInfo, writeFluxome, writeFluxome`

**Methods inherited from class sbrt.shell.mng.AbstractProcessManager**

`check, closeFile, closeRm, execute, getFileFormat, getFileFormat, getFileNameSource, getIterations, getSeed, getTextFileFormat, getTextFileFormat, getZeroCutoff, getZeroCutoff, parseInt, parseLong, printProgress, printProgress, printProgress, runProcess, setInput`

**Methods inherited from class java.lang.Object**

`clone, equals, finalize, getClass, hashCode, notify, notifyAll, toString, wait, wait, wait`

**Methods inherited from interface sbrt.shell.mng.ProcessManager**

`check, execute, setInput`

**Field Detail**

### requiredKeywords

```
public static final UnmodifiableSet<java.lang.String> requiredKeywords
```

:   The set of keywords required to be present in the `ManagerInput`
    objects supplied to instances of this class.

---


### optionalKeywords

```
public static final UnmodifiableSet<java.lang.String> optionalKeywords
```

:   The set of keywords required to be present in the `ManagerInput`
    objects supplied to instances of this class.


**Constructor Detail**

### CdHarFluxVectorManager

```
public CdHarFluxVectorManager()
```


**Method Detail**

### getComputer

```
public static CdHarFluxVectorComputer getComputer(ManagerInput<java.lang.String,java.lang.String> input,
                                                  java.io.PrintWriter out)
```

:   Creates and returns a coordinate-direction hit-and-run flux vector
    computer using the provided input.

    :   **Parameters:**: `input` - the input containing all necessary values.: `out` - the print writer to which status messages will be printed. **Returns:**: a coordinate-direction hit-and-run flux vector computer using the provided input. **See Also:**: `RandomFluxVectorManager.getFluxome(ManagerInput, PrintWriter)`, `FbaManager.getConstraintTolerance(ManagerInput)`, `getComputer(ManagerInput, PrintWriter, IrrevRxnFluxome, double)`

---


### getComputer

```
public static CdHarFluxVectorComputer getComputer(ManagerInput<java.lang.String,java.lang.String> input,
                                                  java.io.PrintWriter out,
                                                  IrrevRxnFluxome fluxome,
                                                  double tolerance)
```

:   Creates and returns a coordinate-direction hit-and-run flux vector
    computer using the provided input.

    :   **Parameters:**: `input` - the input containing all necessary values.: `out` - the print writer to which status messages will be printed.: `fluxome` - the fluxome with which the computer is created.: `tolerance` - the constraint tolerance. **Returns:**: a coordinate-direction hit-and-run flux vector computer created from the provided input. **See Also:**: `FbaManager.getFluxomeSolution(ManagerInput, PrintWriter, Fluxome)`, `AbstractProcessManager.getSeed(ManagerInput, PrintWriter)`, `CdHarManager.getMinChordLength(ManagerInput, PrintWriter)`

---


### getFluxVector

```
public static java.util.Map<java.lang.String,java.lang.Double> getFluxVector(ManagerInput<java.lang.String,java.lang.String> input,
                                                                             java.io.PrintWriter out,
                                                                             Fluxome fluxome,
                                                                             double tolerance)
```

:   Opens the file denoted by `CdHarManager.INITIAL_POINT_FILE` in the
    provided manager input and returns the flux vector it contains.

    :   **Parameters:**: `input` - the input containing a value for `INITIAL_POINT_FILE`.: `out` - the print writer to which a status message will be printed.: `fluxome` - the fluxome to which the flux vector corresponds.: `tolerance` - the amount by which fluxes in the vector are allowed to violate their defined constraints. **Returns:**: the flux vector contained in the file indicated by `CdHarManager#INITIAL_POINT_FILE` in the provided input. **See Also:**: `RandomFluxVectorManager.getFluxVector(String, Fluxome, double)`

---


### getMultiFluxVectorOutputFile

```
public static MapOutputFile<java.lang.String,java.lang.Double> getMultiFluxVectorOutputFile(ManagerInput<java.lang.String,java.lang.String> input,
                                                                                            java.io.PrintWriter out,
                                                                                            Fluxome fluxome)
```

:   Returns an empty output file that is suitable for storing multiple flux
    vectors. The values for `AbstractProcessManager.OUTPUT_FILE_NAME`,
    `AbstractProcessManager.DATA_HEADERS`, and
    `AbstractProcessManager.FILE_FORMAT` in the provided input are
    parsed and used to call the method
    `FbaManager.getMultiFluxVectorOutputFile(String, FileFormat, Set, Fluxome)`.

    :   **Parameters:**: `input` - the input containing values for the listed keywords.: `out` - the print writer to which status messages will be printed.: `fluxome` - the fluxome for which the data headers correspond. **Returns:**: an empty output file that is suitable for storing multiple flux vectors. **See Also:**: `FbaManager.getRxnNameHeaders(ManagerInput, Fluxome)`, `AbstractProcessManager.getFileFormat(ManagerInput)`

---


### interpret

```
public static ApplicationException interpret(java.lang.RuntimeException e)
```

:   Attempts to interpret the provided RuntimeException.

    :   **Parameters:**: `e` - the exception to interpret. **Returns:**: the interpretted exception. **Throws:**: `java.lang.RuntimeException` - the orignal exception if it could not be interpretted.

---


### interpret

```
public static ApplicationException interpret(DimensionException e)
```

:   Returns an `ApplicationException` whose message describes the error
    that ocurred.

    :   **Parameters:**: `e` - a dimension exception. **Returns:**: an `ApplicationException` wrapping the provided exception.

---


### interpret

```
public static ApplicationException interpret(InfeasibleSolutionException e)
```

:   Returns an `ApplicationException` whose message describes the error
    that ocurred.

    :   **Parameters:**: `e` - an infeasible solution exception. **Returns:**: an `ApplicationException` wrapping the provided exception.

---


### interpret

```
public static ApplicationException interpret(UnboundedVariableException e)
```

:   Returns an `ApplicationException` whose message describes the error
    that ocurred.

    :   **Parameters:**: `e` - an unbounded variable exception. **Returns:**: an `ApplicationException` wrapping the provided exception.

---


### interpret

```
public static ApplicationException interpret(InvalidFluxVectorException e)
```

:   Returns an `ApplicationException` whose message describes the error
    that ocurred.

    :   **Parameters:**: `e` - an invalid flux vector exception. **Returns:**: an `ApplicationException` wrapping the provided exception.

---


### interpret

```
public static ApplicationException interpret(ExceededLimitException e)
```

:   Returns an `ApplicationException` whose message describes the error
    that ocurred.

    :   **Parameters:**: `e` - an exceeded limit exception. **Returns:**: an `ApplicationException` wrapping the provided exception.

---


### getRequiredKeys

```
public UnmodifiableSet<java.lang.String> getRequiredKeys()
```

:   Returns the set of keywords the provided `ManagerInput` object must
    contain.

    :   **Returns:**: the set of keywords the provided `ManagerInput` object must contain.

---


### getOptionalKeys

```
public UnmodifiableSet<java.lang.String> getOptionalKeys()
```

:   Returns the set of keywords the provided `ManagerInput` object does
    not necessarily have to contain.

    :   **Returns:**: the set of keywords the provided `ManagerInput` object does not necessarily have to contain.

---


### setInput

```
public void setInput(ManagerInput<java.lang.String,java.lang.String> input,
                     java.io.PrintWriter out)
```

:   Uses the provided input to prepare this process manager for execution. As
    the input is processed, informative messages are printed to the provided
    print writer.

    :   **Parameters:**: `input` - all of the information required to prepare this process manager for execution.: `out` - the print writer to which all messages will be printed.

---


### getProcess

```
public CdHarFluxVectorProcess getProcess()
```

:   Returns the process this process manager controls.

    :   **Returns:**: the process this process manager controls. **Throws:**: `java.lang.IllegalStateException` - if no input was provided to this process manager via `AbstractProcessManager.setInput(ManagerInput)` or `setInput(ManagerInput, PrintWriter)`

---


### execute

```
public void execute(java.io.PrintWriter out)
```

:   Executes the process this process manager controls, and writes the status
    or results of this process to the provided print writer.

    :   **Parameters:**: `out` - the print writer where status messages will be printed. **Throws:**: `java.lang.IllegalStateException` - if no input was provided to this process manager via `AbstractProcessManager.setInput(ManagerInput)` or `setInput(ManagerInput, PrintWriter)`


---


|  |  |  |
| --- | --- | --- |
| **Overview** \| **Package** \| **Class** \| **Use** \| **Tree** \| **Deprecated** \| **Index** \| **Help** | |  |
| **PREV CLASS**   **NEXT CLASS** | **FRAMES**    **NO FRAMES**     **All Classes** |  |
| SUMMARY: NESTED \| FIELD \| CONSTR \| METHOD | DETAIL: FIELD \| CONSTR \| METHOD |  |


---
